# The role of chromosome variation in the speciation of the red brocket deer complex: the study of reproductive isolation in females

**DOI:** 10.1186/1471-2148-14-40

**Published:** 2014-03-04

**Authors:** Marina Suzuki Cursino, Maurício Barbosa Salviano, Vanessa Veltrini Abril, Eveline dos Santos Zanetti, José Maurício Barbanti Duarte

**Affiliations:** 1 NUPECCE - Núcleo de Pesquisa e Conservação de Cervídeos, Departamento de Zootecnia, FCAV -Faculdade de Ciências Agrárias e Veterinárias, UNESP– Universidade Estadual Paulista, CEP 14884-900, Jaboticabal, SP, Brazil; 2 Programa de Pós-graduação em Medicina Veterinária, Reprodução Animal, FCAV, UNESP, CEP 14884-900 Jaboticabal, SP, Brazil; 3 Laboratory of Embryology and Biotechniques of Reproduction, Faculty of Veterinary Medicine, Postal 15004, 91501-970 Porto Alegre, RS, Brazil

**Keywords:** Cryptic species, Hybrids, Chromosomal polymorphism, *Mazama americana*

## Abstract

**Background:**

The red brocket deer, *Mazama americana*, has at least six distinct karyotypes in different regions of South America that suggest the existence of various species that are today all referred to as *M. americana*. From an evolutionary perspective, the red brockets are a relatively recent clade that has gone through intense diversification. This study sought to prove the existence of post-zygotic reproductive isolation in deer offspring between distinct chromosome lineages. To achieve this, inter-cytotype and intra-cytotype crosses were performed, which resulted in both F1 hybrid (n = 5) and pure offspring (n = 3) in captivity.

**Results:**

F1 females were analyzed in terms of their karyotypes, ovarian histology, estrous cycles and *in vitro* embryo production. Pure females presented parameters that were similar to those previously reported for *M. Americana*; however, the parameters for hybrid females were different. Two hybrids were determined to be sterile, while the remaining hybrids presented characteristics of subfertility.

**Conclusions:**

The results support the existence of well-established reproductive isolation among the most distant karyotype lineages and elucidates the need to define all karyotype variants and their geographical ranges in order to define the number of species of red brocket.

## Background

Among mammals, the Cervidae family is known for its wide chromosomal diversification and this is also true for the genus *Mazama*. This genus is considered one of the most complex [1], together with the genus *Muntiacus*[2]. The ancestral forms of the red *Mazama* came into South America approximately 2.5 million years ago, and from that time branched out into various species (*M. bororo, M. nana, M rufina, M. americana*) [1,3,4]. Following cytogenetic studies, the ancestral karyotype of the Cervidae was defined as 2n = 70 and FN = 70 [5,6].

The red brocket group accumulated multiple centric and tandem fusions leading to distinct karyotypes [2], such as 2n = 36 to 41 + 1-6B and FN = 58 in *M. nana*[7] and independently, 2n = 34 + 4-5B and FN = 46 in *M. bororo*[3]. Moreover, the genus presents high intraspecific and interspecific chromosomal polymorphism [4,5,8-10].

In the case of *M. americana*, current studies discuss the possibility of a cryptic species complex due to the significant intraspecific chromosomal variation [3,4]. Significant differences in the patterns of karyotypic evolution in *M. americana* provide strong evidence that the Central and South American lineages are really different species. *M. temama* from Mexico, previously classified as *M. americana temama*, presented a karyotype with 2n = 50, XX/XY and FN = 72 [8].

In Brazil, *M. americana* has a wide geographical distribution ranging from the North to the South of the country [10], and specimens from different geographical locations present high levels of genetic differentiation and diversification in haplotypes and karyotypes [4]; however, they are morphologically reported as a single species with no taxonomic subdivisions [11].

The karyotypes of *M. americana* (species complex) seem to be derived from a single common ancestral karyotype with 2n = 52/53 + 3-4B, XX/XY1Y2 and FN = 54, which arose after successive tandem fusions and an X-autosome fusion [5,9]. In Brazil, the species has been divided into six distinct cytotypes: Rondônia (Ro: 2n = 42♀-43♂/NF = 49), Juína (Ju 2n = 43-44♀/44-45♂ + 3-6B and FN = 48), Jarí (Ja: 2n = 49♂/NF = 56), Carajás (Ca: 2n = 50♀-51♂/NF = 54), Santarém (Sa: 2n = 51♂/NF = 56) and Paraná (Pr: 2n = 52♀-53♂/NF = 56) [12].

**Figure 1:**
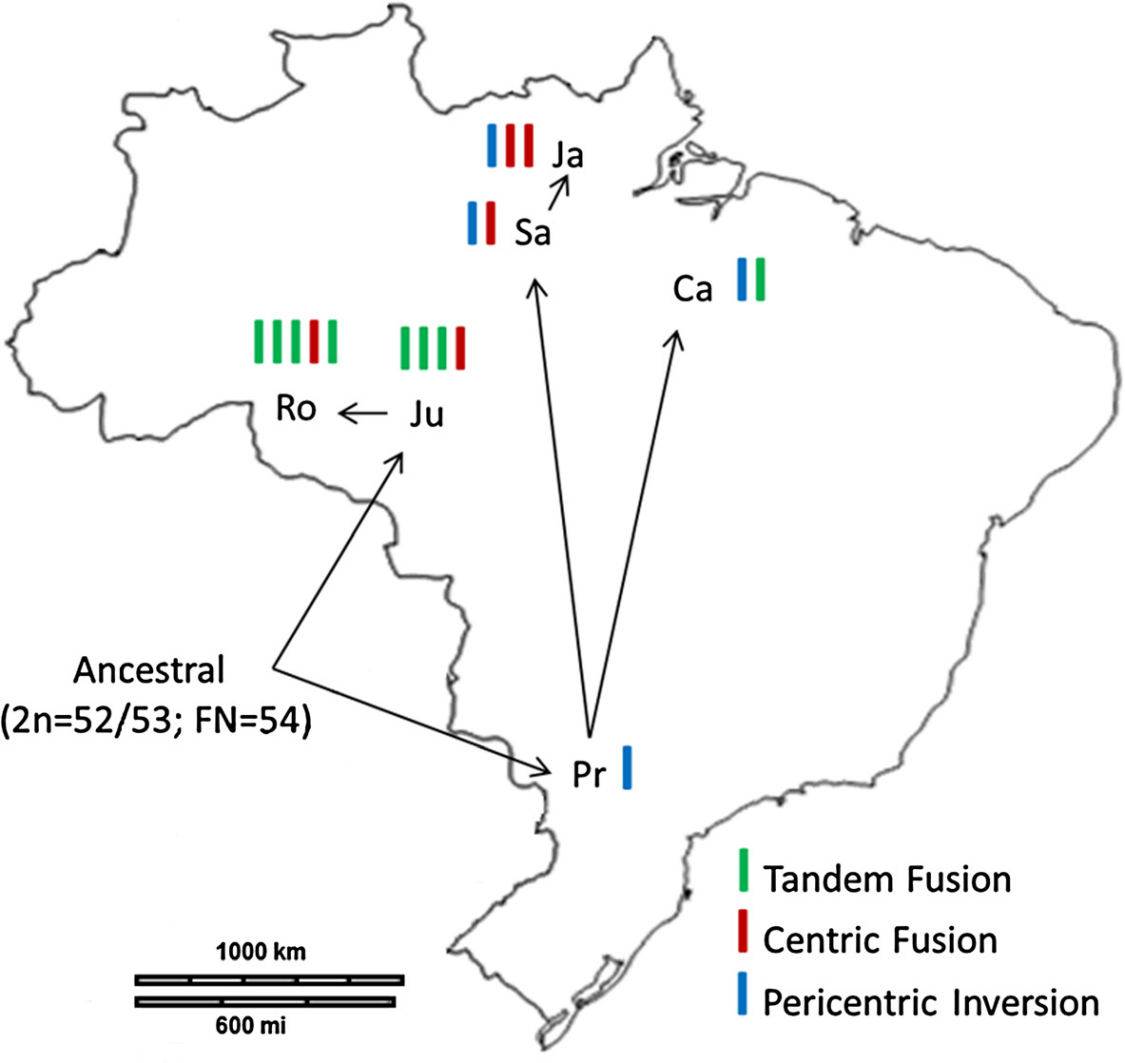
**Chromosomal evolution network.** Relationships of the 6 cytotypes of M. americana analyzed and their geographical distribution, modified from Abril *et al*. [12]. The ancestral karyotype originated the Linage A) (northwest of Brazil) and Linage B) (south and north of Brazil). The chromosomal differentiation of each cytotype from the common ancestor (2n=52-53; FN = 54) was achieved by the fixation of different rearrangements: Linage A is constitute by Ju: Juína (2n=44/45; FN=48) a centric-fusion translocation (red bar) and three tandem-fusion translocations (green bar), and Ro: Rondônia (2n=42/43; FN=46) a rearrangement of the Juína cytotype with another tandem-fusion translocation (green bar). The Linage B is constitute by Pr: Paraná (2n=52/53; FN=56) a pericentric inversion (blue bar) from the ancestral karyotype; Ca: Carajás (2n=50/51; FN=54) a rearrangement of the Paraná cytotype with another tandem-fusion translocation (green bar); Sa: Santarém (2n=50/51; FN=56) a rearrangement of the Paraná cytotype with another centric-fusion translocation (red bar); and Ja: Jarí (2n=48/49; FN=56) a rearrangement of the Santarém cytotype with another centric-fusion translocation (red bar). 2n = diploid number / FN = fundamental number. The colors bars indicate the chromosome rearrangements accumulated by the cytotype.

Another recently discussed point regarding the taxonomy of the species was the discovery of two chromosome lineages: Lineage A, which includes the Rondônia and Juína karyotypes; and Lineage B, which includes the Jarí, Carajás, Santarém, and Paraná karyotypes [12]. Both lineages evolved from a common ancestor through different chromosomal rearrangements and present a high level of genetic differentiation and distance, which led Abril et al. to suggest the existence of two or more distinct species [12]. The chromosomal differentiation of each cytotype from the common ancestor (2n = 52-53; FN = 54) was achieved by the fixation of different rearrangements (Figure 1): i) Paraná, pericentric inversion; ii) Carajás, a rearrangement of the Paraná cytotype with another tandem-fusion translocation; iii) Santarém, a rearrangement of the Paraná cytotype with another centric-fusion translocation; iv) Jarí, a rearrangement of the Santarém cytotype with another centric-fusion translocation; v) Juína, a centric-fusion translocation and three tandem-fusion translocations; and vi) Rondônia, a rearrangement of the Juína cytotype with another tandem-fusion translocation [12].

Most of the advances in chromosomal rearrangement, speciation and their relationship, have been theoretical, especially in mammals [13]. In general, models of chromosome speciation have the same point of view: the reduction of gene flow through the accumulation of chromosomal differences between the progenitor and their descendants that leads to impaired fertility or viability of interspecific hybrids [14]. Animals that are heterozygous for chromosome rearrangements may present anomalous pairings during meiosis, which results in failure during gametogenesis and unbalanced gamete production, both of which cause diminished fertility or even sterility in the organism [15].

One of the most common types of structural rearrangement between species or chromosomal races is the centric fusion or Robertsonian fusion [16]. Individuals heterozygous for a single centric fusion may present diminished reproductive capacity, even though the trivalent structures formed segregate normally during meiosis [17]. On the other hand, a hybrid individual, descendant of progenitors that have accumulated different Robertsonian fusions, can present infertility. This occurs in the *Mus musculus* complex, due to the accumulation of centric fusions between karyotypic races, hybrids present pentavalent structures during meiosis, which leads to the formation of unbalanced gametes and the reproductive isolation of the neospecies [18].

Tandem fusions follow the same pattern as centric fusions, i.e. they cause diminished fertility or a reduction in the fitness of the hybrid, which can lead to reproductive isolation due to the accumulation of rearrangements [19]. Tandem fusions appear to have a special role in the evolution of certain taxa, such as bovids [20]. The difference between swamp buffalo and river buffalo is a single tandem fusion, involving chromosomes 4 and 9 of the river buffalo karyotype [21]. A bull was described as exhibiting a 10% reduction in fertility due to a single tandem fusion [22]. In muntjac deer, 17 tandem fusions and three centric fusions differentiate the Chinese muntjac (2n = 46) from the Indian muntjac (2n = 6♀/ 7♂) [23].

Another chromosome rearrangement that can be involved in speciation is chromosome inversion. Some models suggest that the presence of inversions can lead to genetic differentiation among species, or even to reproductive isolation in populations with gene flow, by reducing recombination between inverted and non-inverted genomic regions [24-26]. In contrast, species that present a high rate of inversion polymorphism, a synaptic adjustment can occurs during meiosis leading to heterosynapsis and chiasma suppression within heterozygous inverted regions and the hybrids are fertile and viable [25,27].

Traditionally, studies involving reproductive isolation seek to prove the presence of sterility or subfertility in hybrids. In order to evaluate the fitness of the female, reproductive parameters such as meiotic parameters in germ cells of fetuses have been used [28-30], together with histological evaluation of the ovaries [30-33] and successful reproduction involving the production of viable fawns [34,35]. Current techniques that produce viable results in a short period of time, such as *in vitro* testing, may also help to infer the reproductive capacity of female hybrids.

The presence of germ cells can be inferred through ovarian activity, which itself is strongly related to the regulation of steroid hormones, such as progesterone and estrogen [36]. A method that is commonly used to evaluate the ovaries of wild animals is the measurement of fecal progesterone metabolite levels (FPM) [37-39]. In the case of Neotropical deer, particularly *M. gouazoubira*, the characteristics of reproductive events have been studied using this method of monitoring the endocrine system [40-43].

The genetic balance of these germ cells can be inferred using an *in vitro* embryo production technique. Only oocytes that have a balanced genetic background are fertilizable and then capable of beginning embryogenesis [44-46].

Based on these discussions surrounding the taxonomy of the red brocket and in light of new reproductive technologies, this study sought to determine the presence or absence of post-zygotic reproductive isolation among the chromosomal lineages of the *Mazama americana* by evaluating the fertility of pure and hybrid females.

## Results

### Hormone measurements

**Figure 2:**
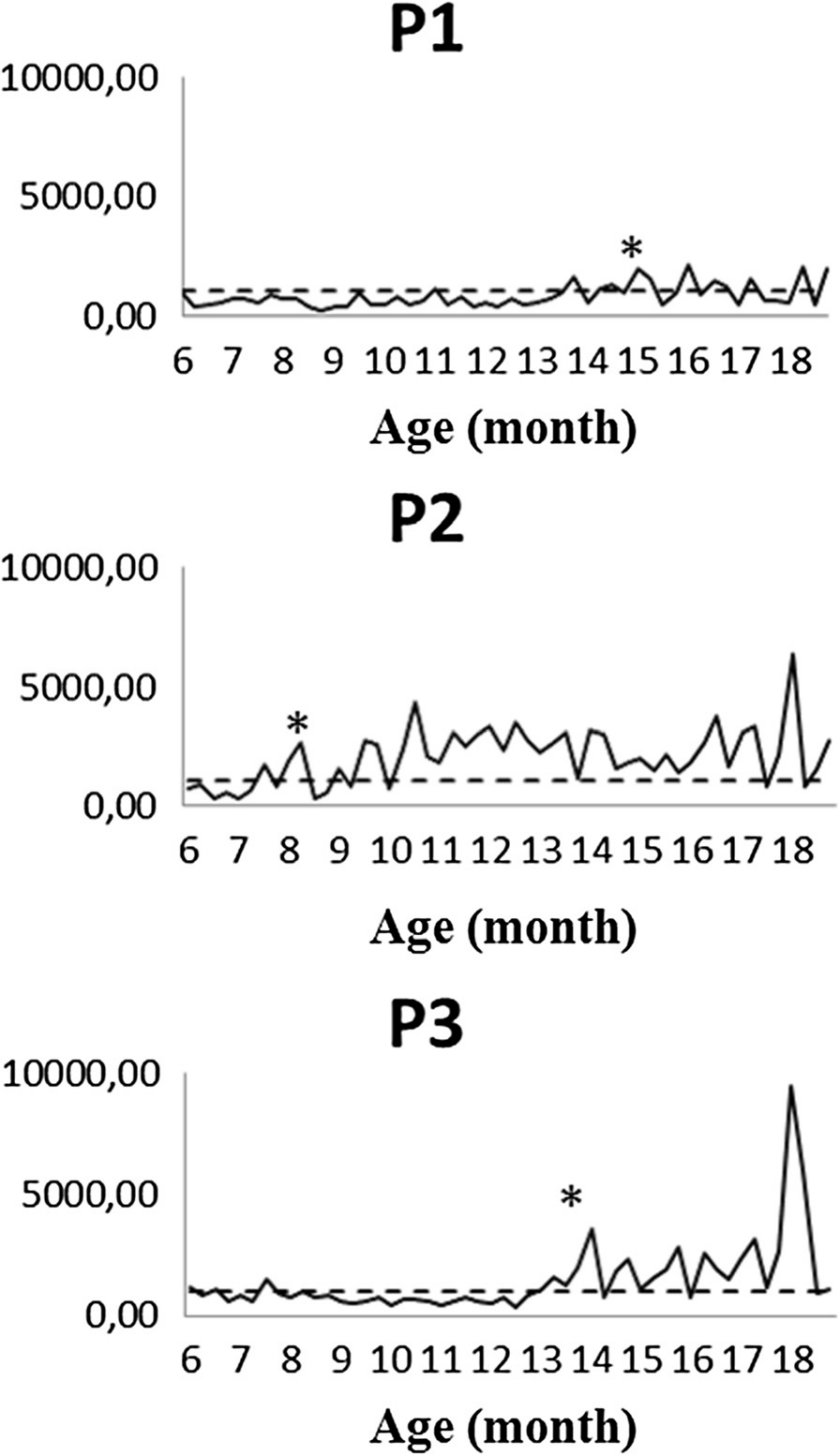
**Hormonal profile of pure females.** Concentrations are expressed in grams of feces/ng of fecal progesterone metabolites (FPM). The dotted line refers to the calculated mean baseline of progestogens. *First ovulation cycle observed.

**Figure 3:**
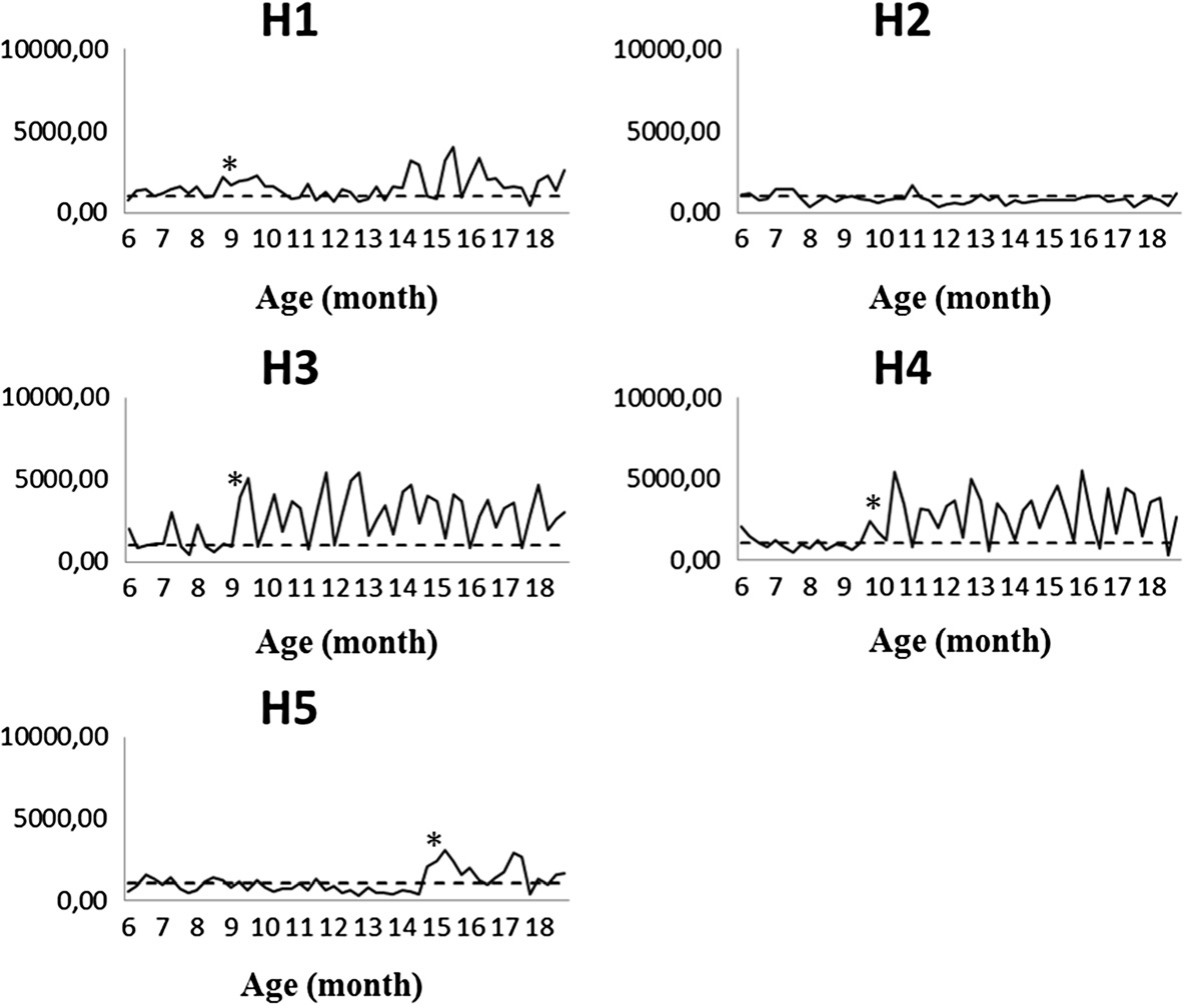
**Hormonal profile of hybrid females.** Concentrations are expressed in grams of feces/ng of fecal progesterone metabolites (FPM). The dotted line refers to the calculated mean baseline of progestogens. *First ovulation cycle observed.

The hormonal profile and the base concentration of FPM were used to determine the onset of puberty (ovarian activity), which varied from eight to 15 months of age (Figures 2 and 3). The females P2, H1, H3, and H4 experienced their first ovulations between eight and 10 months of age, while the females P1, P3, and H5 experienced the onset of puberty later, between 14 and 15 months of age. Two females did not show a cyclic profile similar to the others: the pure female P1, in which the onset of puberty was observed; and the hybrid female H2, which did not show any ovarian activity until 18 months-old.

### Histology of the ovaries

**Figure 4:**
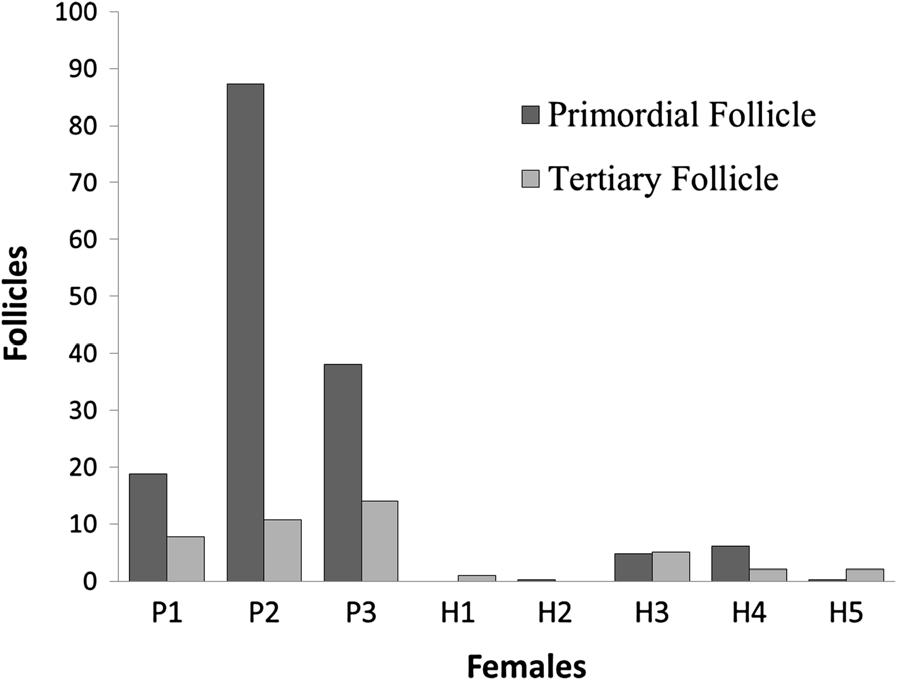
**Number of follicles from histology of the ovaries of *Mazama americana *females.** Number of primordial and tertiary follicles of hinds: Pure (P1, P2 and P3) and Hybrids (H1, H2, H3, H4 and H5).

Both the pure and hybrid females presented follicles in various stages of development, along with corpora lutea and corpora albicans; however, the hybrid females presented a smaller number of follicles than the pure females. Crossings between males from Lineage B (Pr and Ca; higher 2n) and females from Lineage A (Ro and Ju; lower 2n) produced sterile females (H1 and H2) that lacked follicular structures. However, crossings between males from Lineage A with females from Lineage B produced subfertile females (H3 and H4) that possessed follicular structures, though fewer than pure females. Despite being the result of a crossing between two cytotypes from the same lineage (Ju x Ro), specimen H5 presented fewer follicular structures and was more similar to the females H1 and H2 (Figure 4).

### Superovulation, follicular aspiration and in vitro production of embryos

Pure females responded to superovulation treatment and provided a total of 26 aspirated oocytes (8.67 ± 3.06 per female). The total amount of oocytes obtained from pure females using the slicing method (involving half an ovary from each female) was 105 (35 ± 9 per female).

**Figure 5:**
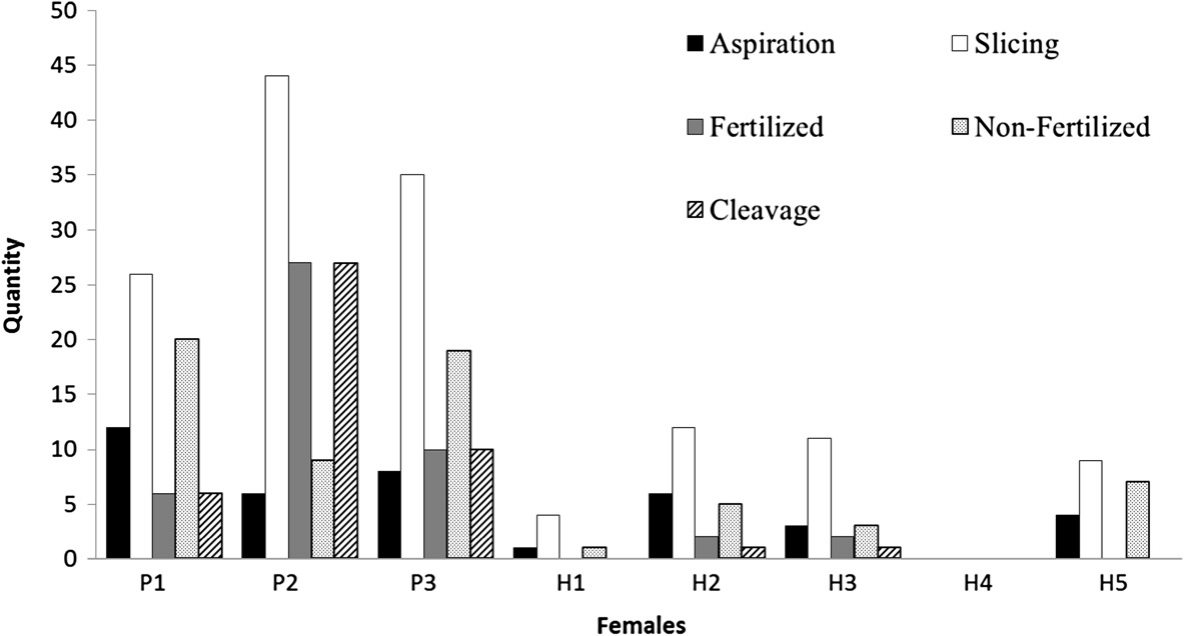
**The total amount of structures obtained from *Mazama americana *females.** Structures obtained by *in vivo* aspiration and *in vitro* embryo production (P - pure females and H - hybrids females). Aspiration: The total amount of oocytes obtained by *in vivo* aspiration. Slicing: The total amount of oocytes obtained using the slicing process. Fertilized: The total amount of embryos fertilized *in vitro*. Non-fertilized: The total amount of embryos not fertilized *in vitro*. Cleavage: The total amount of embryos that underwent cleavage (2–16 cells).

All of the oocytes were forwarded for maturation, fertilization, culture medium and development *in vitro*. Hoechst staining verified that 32.82% (43 out of 131) of the embryos experienced cleavage, 36.64% (48 out of 131) were considered unfertilized oocytes and 30.53% (40 out of 131) presented inconclusive structures. Development was halted in all of the embryos before the blastocyst stage, but we were still able to obtain five morulae with more than 16 cells (Figure 5).

**Figure 6:**
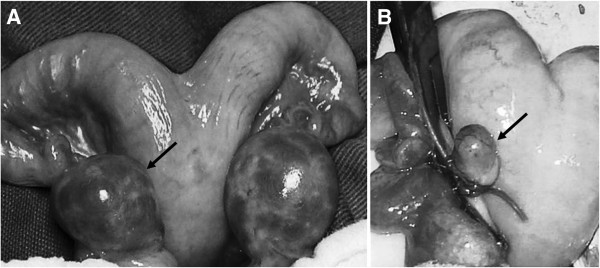
**Superovulation response. A** - The ovary of pure female P3, which responded to superovulation treatment with follicular development (arrow). **B** - The ovary of female H1, which did not respond to superovulation treatment (arrow).

The female hybrids H1 and H2 did not respond to superovulation treatment and showed no follicular development (Figure 6), while females H3, H4 and H5 developed follicles. Fourteen oocytes were obtained by aspiration (2.8 ± 5.6 per female), and 36 oocytes (7.2 ± 11.57 per female) were obtained using the slicing method on half of each ovary. Of these oocytes, 37 were forwarded for maturation, and 36 remained for fertilization, culture medium and development *in vitro*.

Hoechst staining revealed an 11.11% rate of fertilization (results that include cases of polyspermy) and a very low rate of cleavage (5.55%). Sixteen of the 37 oocytes (44.44%) were considered unfertilized, and 16 (44.44%) produced inconclusive results. Development was halted in all of the embryos before the morula stage; division of only four to six cells was observed, even in the pronucleus stage.

## Discussion

### Reproductive abilities

The reproductive parameters analyzed in this study (FPM measurement, ovarian histology, response to superovulation and the production of embryos *in vitro*) provide conclusions concerning the reproductive abilities of pure females. The research methods used herein determined luteal phase profiles, the presence of ovarian structures, a satisfactory response to superovulation and embryos produced *in vitro*.

The hormone profile of FPM from the pure females indicated the presence of luteal phases, which represent ovarian activity [40,47-50], as well as the absence of reproductive seasonality in these animals [51,52]. Most female Cervidae experience their first ovulation at approximately one year of age, while females from smaller species can expect their first ovulation sooner [53]. In this study, important variations occurred in the age at which the onset of puberty was experienced; in some cases this was very different from the onset at 11 months-old, as previously cited for this species [51].

Changes to the onset of puberty can be triggered by different factors. The rapid weight gain experienced by animals in captivity [53,54], which increases leptin levels in the blood [55-58], can anticipate the onset of puberty. Similarly, the stress of captivity can delay puberty because of the interference of glucocorticoids in the gonadal axis [59-61].

Among the females that experienced puberty earlier (between 8 and 10 months), only one was pure (P2); the other were hybrids and they eventually cycled. In contrast, the hybrid H5 and the pure females P1 and P3 experienced the onset of puberty at 14 months-old.

Female H2 lacked an ovulation cycle, results which can indicate sterility, particularly sterility associated with the lack of ovarian structures. This absence of ovarian structures was also observed in the female H1. The progenitors of females H1 and H2 are carriers of complex chromosome rearrangements (♂Pr: one pericentric inversion; ♀Ju: one centric fusion and three tandem-fusion translocations).

Histological sections from the other female hybrids (H3, H4 and H5) presented germ cells (primordial follicles), though the average number of these structures was lower compared with pure females. Thus, the number of ovarian structures indicates subfertility (H3; H4 and H5) or sterility (H1 and H2) in the hybrid hind studied. The ovarian activity determined in the histological evaluation is related to the response to the superovulation treatment performed on the females; for this reason, a large number of tertiary follicles were obtained, particularly from the pure females.

As expected, the rates of oocyte retrieval *in vivo*, even following FSH treatment, were lower in hybrid females than in pure females. These results reflect a satisfactory response to the superovulation protocol used. In wild ruminants, the rates of oocyte retrieval are similar: 9.3 ± 1.7 oocytes were retrieved from the species *Gazella dama mhorr*[62], and 3.21 ± 0.51 were retrieved from the species *Cervus elaphus*[63]. For the species *M. gouazoubira*, an average of 10.4 ± 1.1 follicles was observed in each hind [64]. In the last study, the same superovulation protocol was used, though oocyte aspiration was not performed.

High rates of cleavage blocking and low rates of embryo production are both common in *in vitro* fertilization studies on ruminants, particularly among wild species (*Gazella dama mhorr*[62], *Cervus nippon* and *Cervus elaphus*[65]), and these factors seem to be related to inadequate medias for development *in vitro*[65-68]. Thus, it is likely that embryonic development up to the morula stage in pure females may be due to these factors, while the blocking of embryos in hybrid females during the initial stages of development is primarily related to the chromosomal imbalance of the embryos.

If the oocyte is not fertilized, the mechanism that initiates cleavage may be activated and oocyte division occurs even in the absence of fertilization; for cleavage to continue, the genome of the embryo must be activated. In mice, it has been reported that genome activation occurs during the second round of cell division [69]. In bovine, activation occurs later, between the two- and eight-cell stage. In sheep and goats, activation occurs between the eight- and 16-cell stage [70]. There are no studies on gene activation in embryos from Cervidae; therefore, the blocking of embryos from hybrid females before the morula stage (between one and 12 cells) could be related to division by parthenogenetic activation or to failed embryonic genome activation. One of the factors that cause embryonic genome activation to fail is chromosome imbalance.

The high percentage of unfertilized oocytes in hybrid females could be due to a failure during the maturation stage. Incompetent oocytes are deficient in the amount of mRNA necessary to promote nuclear and cytoplasmic maturation. This deficiency impedes the penetration of spermatozoa and, consequently, embryonic development [71]. A study of the synaptonemal complex in fetuses from female hybrids of different species of wallabies determined irregularities in chromosome pairing during the first phase of meiosis. During this phase, unpaired regions and multiple bonds (polyvalence) were identified, among other irregularities [72]. This abnormal meiotic division of the oocyte leads to the production of genetically imbalanced gametes that are unable to continue meiosis or produce the amount of mRNAs necessary for their maturation and the development of reproductive abilities; for this reason, embryonic production in hybrids fails.

### Post-zygotic reproductive isolation

The data obtained in this study makes it possible to draw certain conclusions regarding the reproductive abilities of pure females and the presence of subfertility and sterility in hybrids. Specimens that were the product of crossings between females from Lineage B (Pr and Ca) and males from Lineage A (Ju and Ro) presented subfertility, while those that were produced from ♀A x ♂B crossings were almost completely sterile. The females H1 and H2 (♀Ju x ♂Pr) were sterile, and were the product of progenitors that carried five chromosomal rearrangements: one pericentric inversion, one centric-fusion translocation, and three tandem-fusion translocations. The female H3 (♀Pr x ♂Ju) was subfertile, even though the differences between her progenitors involved the same chromosomal rearrangements as the females H1 and H2.

The other two females that were subfertile were H4 (♀Ca x ♂Ju) and H5 (♀Ju x ♂Ro). The female H4 presented the greatest chromosomal difference among the parental cytotypes: one pericentric inversion, one centric fusion and four tandem fusions. The parental cytotypes of female H5 differ by only one tandem fusion. Even though they are carriers of a heterozygous centric fusion involving the same chromosome pairs, female H5 did not inherit this centric fusion from its progenitors. Thus, its subfertility, detected by reproductive parameters, is related to the difference that the tandem fusion generates between the Ju and Ro cytotypes. This type of rearrangement seems to have an important role in the origin of fertility problems and in the reproductive isolation of several species [19-23].

The accumulation of chromosomal rearrangements leads to infertility due to the death of germ cells, which in turn is due to failures during meiotic pairing [14,73,74]. This conclusion seems to be the most likely explanation for the reproductive impairment observed in the female hybrids. In hybrids, the chromosomal rearrangements (pericentric inversion, centric-fusion translocations and tandem-fusion translocations) are heterozygous and could be the cause of the chromosome imbalance that led to subfertility and sterility [14,75]. The accumulation of chromosomal rearrangements that are involved in the differentiation between Lineages A and B of *M. americana* may be the cause of the reproductive isolation identified in our experiment.

Because the cytotypes were geographically isolated [2], chromosomal differentiation between the populations seems to have occurred allopatrically [76]. Chromosome fragility and a high rate of chromosomal rearrangements are probably involved in the karyotype evolution of Cervidae and both of these would explain the system of *Mazama americana* cytotypes [77]. According to Duarte et al. [4], the divergence of the most distant lineages of *M. americana* most likely occurred 2.5 million years ago; a relatively short amount of time for two such karyotypically different species to develop. Two species that diverged from this branch were *M. bororo* and *M. nana*, with 2n of 32 and 38, respectively [3,7]. Thus, chromosomal rearrangements seem to be the most important and efficient mechanism of speciation in this group to achieve the generation of reproductive isolation after such a short period of time.

Villena and Sapienza [78] argued that karyotype evolution in mammals has occurred mainly through the non-random segregation of chromosomes during meiosis in the oocyte. They affirmed that factors that regulate female meiosis play a role in the fixation of certain chromosomal rearrangements. In the case of the genus *Mazama*, the main rearrangements were those that reduced the diploid number in populations, such as centric-fusion and tandem-fusion translocations, which are both present in large quantities during the differentiation between the cytotypes of *M. americana*[12,79].

In 2008, Duarte et al. [4] evaluated the genetic distance between the *M. americana* cytotypes. They suggested that the separation of the cytotypes may have occurred at the end of the Pliocene, after the great migration of the ancestral forms of these species, which came from North America. After the isolation of the populations, both genetic and cytogenetic diversification occurred. Based on cytogenetic analyses, Abril et al. [12] suggested the differentiation of two clades of the species *M. americana*, which is supported by molecular analyses of mitochondrial DNA. These analyses revealed that the Rondônia and Juína cytotypes compose the A clade, with a smaller chromosomal number, and that the Paraná, Carajás, Jarí and Santarém cytotypes compose the B clade, with a larger diploid number. Despite chromosomal and genetic differences, the populations maintained the same phenotype, and can therefore be considered as cryptic species [80].

All of the crossings performed between Lineages A and B resulted in reproductive isolation, supporting the separation of these two lineages into at least two species. Since few crossings of deer with distinct karyotypes were obtained from the same chromosomal lineage (one female; H5), it is difficult to prove the existence of reproductive isolation within chromosome lineages, despite the fact that the hind produced was subfertile.

The high degree of chromosomal divergence in *M. americana* causes an important taxonomic problem. Rossi’s morphological studies [11] did not identify any significant differences between populations of *M. americana*. Thus, though there are relevant and consistent chromosomal and molecular differences among the populations [12], including the well-established case of reproductive isolation, these differences are not reflected in morphological differences. This inconsistency makes it difficult to identify species without using genetic methods.

Despite the existence of well-defined cytotypes that have been proven to be reproductively isolated, questions remain regarding the taxonomic level of the closest cytotypes, such as Paraná and Carajás, or even Juína and Rondônia. These uncertainties cause problems that are reflected in conservation and raise the question whether each cytotype should be considered independent from a taxonomic perspective. A larger sample must be collected, and more studies, particularly concerning the existence of reproductive isolation among closely related cytotypes must be performed so that the correct management and conservation decisions can be made. One of the cytotypes, that which is found in the Atlantic Forest region of South America, could be considered the most threatened of the cervid species in the Americas if it is considered to be from a different taxon than the remaining red brocket deer.

## Conclusion

In this study, we verified that two of the six chromosome variants that exist within *M. americana* (Lineage A- Paraná and Lineage B- Juína) possess an efficient mechanism of post-zygotic reproductive isolation, which involves infertility or subfertility of the hybrid. Once the true impossibility of gene flow between the lineages is identified, more concrete discussions on modifications to the taxonomy of this species can be initiated. In light of the results of this study, combined with previous studies, both lineages may be considered as cryptic species that present the same phenotype. Populations with similar karyotypes must be evaluated more carefully, since there is a reasonable likelihood that they are also distinct species. Chromosomal changes have proven to be an efficient and powerful mechanism in the isolation of populations and in the formation of species in the genus *Mazama*.

## Methods

The present study was approved by the Animal Ethics and Welfare Committee (*Comitê de Ética e Bem-estar Animal*, CEBEA) of the School of Agricultural and Veterinary Sciences (*Faculdade de Ciências Agrárias e Veterinárias*, FCAV) of São Paulo State University (UNESP), Jaboticabal, SP, Brazil.

### Animals

The females used in the experiment were generated by crossing deer from the species *M. americana*. The animals belonged to the Deer Research and Conservation Center (*Núcleo de Pesquisa e Conservação de Cervídeos – NUPECCE*) of the Department of Animal Science of the FCAV-UNESP in Jaboticabal, Sao Paulo, Brazil. The parents were karyotyped and identified as members of different cytotypes: Juína, or Ju (2n = 43-44♀/44-45♂ + 3-6B and FN = 48); Rondônia, or Ro (2n = 42♀/43♂ + 3-5B and FN = 46); Paraná, or Pr (2n = 52♀/53♂ + 3-4B and FN = 56); and Carajás, or Ca (2n = 50♀/51♂ + 3-4B and FN = 54). The deer with the most similar karyotypes were considered to be from the same chromosome lineage. Thus, two lineages were established: Linage A) Ju and Ro cytotypes and Lineage B) Pr and Ca cytotypes.

Only female offspring were used in this study. From the crossings between specimens of the same cytotype (considered “pure,” or P), two females from the Juína cytotype were obtained (P1 and P2), as well as one female from the Rondônia cytotype (P3). From the crossings between specimens with different cytotypes (considered hybrids, H) five females were obtained: crossings between the Juína and Paraná cytotypes (H1 and H2 = ♂Pr x ♀Ju; H3 = ♂Ju x ♀Pr), Carajás and Juína (H4 = ♂Ju x ♀Ca) and Juína and Rondônia (H5 = ♂Ro x ♀Ju).

### Measurement of fecal progesterone metabolites (FPM) levels

#### Feces collection

The feces samples used for measuring hormones were collected over the course of a year, with seven days between each collection. Collections began when the specimens were six months-old, and they were completed when each hind reached 18 months of age. Collection did not begin until the hind had been weaned (six months of age), because until this period, the fawn remained in the female’s stall, which made it difficult to collect individual fecal matter. The samples were stored in plastic bags and maintained at -20°C until processing was begun.

#### Processing the samples

The samples were dried in an oven at 56°C for approximately 72 h and then pulverized [49,81]. The metabolites were extracted from the fecal samples as described by Graham [50]. The supernatant was separated and stored at -20°C until the measurements were performed.

#### Enzyme immunoassay (EIA)

For the measurements determined by an EIA (Multiskan MS, Labsystem, Helsinki, Finland), CL425 monoclonal antibody (CJ Munro, University of California, Davis, USA) was used for progestogens [50]. All fecal extracts were diluted (1:256) in a dilution buffer and measured in duplicate. The hormone measurements were validated using the process described by Brown [82]: (1) parallelism between serial dilutions from a pool of fecal extracts and a standard curve; and (2) significant recovery of exogenous progesterone added to fecal extracts (y = -0.0231x + 2.5316 and R^2^ = 0.9797). The interassay coefficient of variation for the two separate controls was 11 (30% binding) and 19.6 (70% binding) for metabolites of fecal progesterone. All of the data collected on the feces were expressed based on its dry weight.

### Estrus synchronization, superovulation and surgical procedure

At 18 months of age, the females were submitted to the estrus synchronization and superovulation protocol. Estrus was synchronized with an intravaginal progesterone insert (CIDR®- *Controlled Internal Drug Release*® - Pfizer®, USA) for 8 days, followed by intramuscular application of 0.25 mL of estradiol benzoate (Estrogin, Farmavet Produtos Veterinarios Ltda, Brazil) at the moment the implant was inserted (D0). On the day four (D4) of implantation of the progesterone insert, the administration of a follicle stimulating hormone (FSH) (Folltropin®-V, Tecnopec, Canada) was initiated: 130 mg divided into 8 equal doses that were applied every 12 hours [83]. Eight days after beginning treatment (D8), the specimens were submitted to a laparotomy in order to perform follicular aspiration of an ovary *in vivo* and an ovariectomy of the ovary that was contralateral to the one that had been aspirated. To perform the surgery, the hind were fasted for 24 h and were then anesthetized with 5.0 mg/Kg of ketamine hydrochloride (Vetaset® - Fort Dodge, Brazil), 0.3 mg/Kg of xylazine hydrochloride (Rompum® - Bayer, Brazil), and 0.5 mg/Kg of midazolam (Dormonid® - Roche, Brazil) and maintained under isoflurane (Forane® - Abbott, Brazil) during the procedure. After the surgery was completed, the intravaginal insert (CIDR®) was removed.

### In vivo oocyte aspiration

The process of *in vivo* aspiration was performed with a number 22 intravenous infusion device (BD®) attached to a 10-mL syringe. The aspirated follicular fluid was maintained in a PBS solution completed with Heparin (10 UI/mL). The solution had been previously heated to 37°C and the fluid was maintained at this temperature until the onset of *in vitro* production (IVP). The ovary that was removed was stored in the same solution, and was later divided into two equal parts: one was used to obtain oocytes by slicing, and the other for a histological exam.

### Ovarian histology

The half of the ovary used for histology was maintained in Bouin solution for fixation. In order to prepare the histological slides, 5 μm sections were made and the slides received two types of stains: HE and Mason’s Trichrome stain. The histological sections were viewed under a light microscope and the morphology of their structures were analyzed descriptively. The number of structures in the entire histological sections was determined using the Axio Vision program, version 4.8.2.

### Semen collection and preparation for in vitro fertilization (IVF)

The semen donors were the fathers of the female donors of the oocytes. Semen collection for IVF was performed using electroejaculation. The semen collected was pre-diluted in Tris-yolk [84,85] and its motility, vigor, and concentration were analyzed. Next, the concentration was adjusted to 50×10^6^ spermatozoa/mL and the semen was stored in 0.25-mL straws and frozen in a TK-3000® portable medical refrigerator (TK Equipamentos para Reprodução, Brazil). It was maintained at -196°C until IVF was performed. Later, one straw was defrosted to 35°C for 20 seconds, and the semen was deposited into a 2-mL tube containing a discontinuous Percoll Gradient (Biotech Pharmacy, Sweden) of 90% and 45% [86] and inserted in drops of an IVF medium (TL-Semen, 500 mg amikacin sulfate, SOF, PHE, Heparin and 176 UI/mg and serum from sheep in estrus).

### In vitro production of embryos

The portion of the ovary used to obtain oocytes was sliced into a PBS solution completed with Heparin (10 UI/ml) and heated to 37°C. Next, the solutions obtained from slicing and from *in vivo* aspiration were analyzed using a stereomicroscope in order to classify the oocytes. The classification process followed the parameters determined for bovine reproduction [87]. Those that were classified as being of higher quality were forwarded for maturation *in vitro* (TCM-199; SFB10%; 0.20 mM pyruvate; 83.3 μg/mL amikacin sulfate; 1.0 μg/mL FSH; and 50 μg/mL hCG) for 27 h [88]. The oocytes were inserted in the IFV medium with semen, and after 18 h of fertilization, the potential embryos were transferred to culture medium (Medium of SOF; 2,5% SFB; 5 mg/mL BSA). On day 10, the pre-embryos were stained with Hoechst 33342 in order to determine the presence or absence of pronuclei and blastomeres, which indicate fertilization and embryonic development, respectively. To achieve this, the oocytes/embryos were transferred into drops (made up of a blocking solution, 10 μL of Hoechst 33342 and glycerol) on a slide and covered with a cover slip. The sides of the slide were sealed with enamel. After 2 h, the analyses were completed under an epifluorescence microscope (excitation filter BP 330-385 nm and barrier filter BA 420), and the samples were photographed using a digital camera (Olympus® C-5060, 5.1 megapixels).

### Results analysis

#### Measurements of FPM

The profiles obtained by FPM measurements were analyzed following the process described by Graham [89]. Data concerning the concentration of all of the female samples were combined to calculate the overall mean FPM concentration. Values that were greater than this mean plus 1.75 SD (standard deviation) were temporarily removed from the data. The mean was recalculated and the process of removing concentrations was repeated until no value exceeded the mean plus 1.75SD. The remaining data were considered to represent the baseline FPM concentrations. The onset of the estrous cycles was considered to be when the FPM concentrations were greater than the baseline mean and remained high for more than two weeks, followed by a drop to baseline levels. The onset of puberty was considered to be when the beginning of the first cycle was evident.

#### Histological structure count

The results are presented as the mean ± SEM (standard error) of the analysis of three histological sections for each hind. The results from the hybrid females and the “pure” females were descriptively compared.

#### Response to superovulation

The superovulation process was evaluated based on the amount of oocytes obtained from the follicular aspiration of both the hybrid and pure females (mean ± SD), and the results were descriptively compared.

#### In vitro production of embryos

Production was evaluated by observing the nuclear Hoechst 33342 staining of the oocytes and embryos. The structures that presented nuclei in different phases of meiosis were considered unfertilized, while the structures that presented pronuclei or blastomere nuclei were considered fertilized. Degenerated oocytes/embryos, fragmented oocytes/embryos, or oocytes/embryos with the presence of too many cumulus cells that prevented the visualization of the nuclei were considered inconclusive. The number of fertilized and unfertilized structures is presented as a percentage, and the results from the hybrid and the pure females were compared.

## Availability of supporting data

The data sets supporting the results of this article are available in the Knowledge Network for Biocomplexity repository, knb.312.1. (http://knb.ecoinformatics.org/m/#view/knb.312.1).

## Competing interests

The authors declare that there are no competing interests.

## Authors’ contributions

MSC conceived and designed the study, participated in the acquisition, analysis and interpretation of data and drafted the manuscript. MBS, VVA and ESZ participated in the study design, acquisition, analysis and interpretation of data and helped to draft the manuscript. JMBD participated in the study design and coordination, and helped to draft the manuscript. All authors read and approved the final manuscript.
